# Pharmacokinetic Properties of Baitouweng Decoction in Bama Miniature Pigs: Implications for Clinical Application in Humans

**DOI:** 10.1155/2024/5535752

**Published:** 2024-05-10

**Authors:** Qianqian Xu, Huilan Gao, Fuqiang Zhu, Wenliang Xu, Yubo Wang, Jinwen Xie, Guangjun Guo, Limei Yang, Li Ma, Zhiqiang Shen, Jichang Li

**Affiliations:** ^1^Shandong Binzhou Animal Science and Veterinary Medicine Academy, Binzhou, China; ^2^College of Veterinary Medicine, Northeast Agricultural University, Harbin, China; ^3^Binzhou Inspection and Testing Center, Binzhou, China; ^4^School of Food and Advanced Technology, Massey University, Palmerston North, New Zealand; ^5^Department of Thoracic Surgery, Binzhou Medical College Hospital, Binzhou, China

## Abstract

Traditional Chinese medicine (TCM) serves as a significant adjunct to chemical treatment for chronic diseases. For instance, the administration of Baitouweng decoction (BTWD) has proven effective in the treatment of ulcerative colitis. However, the limited understanding of its pharmacokinetics (PK) has impeded its widespread use. Chinese Bama miniature pigs possess anatomical and physiological similarities to the human body, making them a valuable model for investigating PK properties. Consequently, the identification of PK properties in Bama miniature pigs can provide valuable insights for guiding the clinical application of BTWD in humans. To facilitate this research, a rapid and sensitive UPLC-MS/MS method has been developed for the simultaneous quantification of eleven active ingredients of BTWD in plasma. Chromatographic separation was conducted using an Acquity UPLC HSS T3 C_18_ column and a gradient mobile phase comprising acetonitrile and water (containing 0.1% acetic acid). The methodology was validated in accordance with the FDA Bioanalytical Method Validation Guidance for Industry. The lower limit of quantitation fell within the range of 0.60–2.01 ng/mL. Pharmacokinetic studies indicated that coptisine chloride, berberine, columbamine, phellodendrine, and obacunone exhibited low *C*_max_, while fraxetin, esculin, fraxin, and pulchinenoside B_4_ were rapidly absorbed and eliminated from the plasma. These findings have implications for the development of effective components in BTWD and the adjustment of clinical dosage regimens.

## 1. Introduction

The Baitouweng decoction (BTWD) is derived from the Treatise on Febrile Diseases authored by Zhongjing Zhang during the Eastern Han Dynasty. It consists of *Pulsatillae radix* (Bai Tou Weng), *Coptidis rhizoma* (Huang Lian), *Phellodendri chinensis cortex* (Huang Bai), and *Fraxini Cortex* (Qin Pi). According to traditional Chinese medicine theory, this decoction exhibits properties such as heat evil clearance, superficial evil expulsion, and blood cooling for diarrhea cessation. In the *Treatise on Febrile Diseases*-*Bianjueyinbingmaizhengbingzhi*, BTWD has historically been employed primarily for the treatment of dysentery accompanied by symptoms of heat, anal prolapse, and swelling. It has long been regarded as the preferred prescription for hygropyretic dysentery, with a history of usage spanning centuries. In contemporary medicine, BTWD is used for the management of digestive system disorders [1, 2]. The chemical constituents present in BTWD encompass alkaloids, coumarins, saponins, limonins, sterols, and lignanoids. Among these constituents, alkaloids and coumarins are believed to constitute the principal material foundation of BTWD. Coptisine chloride, berberine, columbamine, phellodendrine, palmatine, obacunone, esculetin, fraxetin, esculin, fraxin, and pulchinenoside B_4_ have been recognized as the primary active constituents, exhibiting a broad range of pharmacological effects including antioxidant [3], anti-inflammatory [4], antigastrointestinal cancer [5, 6], hepatic fibrosis amelioration [7], gastroprotective [8], and intestinal epithelial barrier protective activity [9].

The pharmacokinetic properties of these constituents in the human body are of utmost importance for clinical investigations. Several studies have reported on the pharmacokinetic properties of these aforementioned constituents using high-performance liquid chromatography (HPLC) [10] or mass spectrometry (MS) techniques [11–16]. Currently, the majority of pharmacokinetic (PK) studies on BTWD primarily focus on a limited number of its components, with the maximum number of chemicals analyzed being seven [14]. These seven components include anemoside B_4_, phellodendrine, berberine, palmatine, obacunone, esculin, and esculetin. However, the analysis did not include coptisine chloride, fraxin, and fraxetin, which are important components of BTWD. Therefore, the current studies are insufficient in providing a comprehensive description of the PK properties of BTWD. Consequently, it is crucial to develop novel analytical methods that can systematically evaluate the pharmacokinetic properties of BTWD.

Currently, the majority of PK studies conducted on BTWD have primarily focused on rats, thereby differing from those conducted on humans. However, Chinese Bama miniature pigs exhibit notable anatomical and physiological resemblances to the human body, rendering them exceptional models for investigating cardiovascular, gastrointestinal, and renal system research [17]. Researchers reported that Bama miniature pigs are suitable for use in drug evaluation studies [18]. Consequently, the PK characteristics identified in Bama miniature pigs hold significant value in informing the clinical application of BTWD in humans.

The objective of this study was to establish a UPLC-MS/MS method that is both rapid and sensitive for the simultaneous quantification of various compounds (coptisine chloride, berberine, columbamine, phellodendrine, palmatine, obacunone, esculetin, fraxetin, esculin, fraxin, and pulchinenoside B_4_) in plasma samples of BTWD. Additionally, this method was used to conduct pharmacokinetic studies on Bama miniature pigs.

## 2. Materials and Methods

### 2.1. Chemicals and Reagents

The reference standards, namely coptisine chloride, berberine, columbamine, phellodendrine, palmatine, obacunone, esculetin, fraxetin, esculin, fraxin, and pulchinenoside B_4_, were procured from the National Institute for Food and Drug Control in Beijing, China, with a minimum purity of 98%. Methanol and acetonitrile of HPLC grade were obtained from Merck in Germany, while formic acid with a minimum purity of 99% was sourced from Anaqua Chemicals Supply in America. Pure water with a resistivity of at least 18.2 MΩ∙cm was generated using a Milli-Q system manufactured by Millipore in Bedford, USA. All other chemicals used in the study were of analytical grade.

*Pulsatillae radix* (originating from Liaoning, China, with batch no. 20181020 and voucher specimen number BTW008) and *Fraxini Cortex* (originating from Liaoning, China, with batch no. 20180126 and voucher specimen number QP012) were procured from Hebei Renxin Pharmaceutical Co., Ltd. *Coptidis rhizoma* (originating from Sichuan, China, with batch no. 20181124 and voucher specimen number HL136) was obtained from Anguo Shenghui Chinese Medicine Yinpian Co., Ltd. *Phellodendri chinensis cortex* (originating from Sichuan, China, with batch no. 20180728 and voucher specimen number HB014) was acquired from Hebei Qiyitang Pharmaceutical Co., Ltd. These samples were subsequently stored in the sample storage room of the Shandong Binzhou Animal Science and Veterinary Medicine Academy. The authenticity and quality of all traditional Chinese medicines used in this study were verified according to the methods outlined in People's Republic of China Veterinary Pharmacopoeia (2020 Edition).

The preparation of BTWD involved combining air-dried *Pulsatillae radix* (30.0 g), *Fraxini Cortex* (24.0 g), *Coptidis rhizoma* (12.0 g), and *Phellodendri chinensis cortex* (24.0 g), followed by extraction with 900 mL of water at 100°C for 1.0 h using a condensing reflux device. This process was repeated twice with 700 mL of water for each extraction, also for 1 h. The resulting extracts were combined and concentrated under reduced pressure using a rotary evaporator at 60°C, resulting in a solution with a concentration of 0.5 g crude herb per 1.0 mL decoction. The solution was then subjected to centrifugation at 3400 × g for 10 mins, and the supernatant was further concentrated to achieve a concentration of 1.0 g crude herb per 1.0 mL decoction. The final solution was stored at −20°C until needed.

### 2.2. Instruments and Analytical Conditions

The LC-MS analysis was conducted using a Waters Acquity UPLC I-Class system (Waters, USA) coupled with a Xevo TQ-XS mass spectrometer equipped with a heated electrospray ionization source. Chromatographic separation was carried out on an Acquity UPLC HSS T3 C_18_ column (2.1 mm × 50 mm, 1.8 *μ*m) from Waters, USA, with a flow rate of 0.4 mL/min and a column oven temperature of 40°C. The mobile phase consisted of 0.1% aqueous formic acid (A) and acetonitrile (B). The gradient elution program was as follows: 0–2.0 min, 10%–60% B; 2.0–2.2 min, 60%–95% B; 2.2–3.2 min, 95% B; 3.2–3.5 min, 95%–10% B; and 3.5–5.5 min, 10% B. Mass spectrometric detection was conducted using both positive and negative ionization modes. The source parameters used were as follows: a spray voltage of 1.00 KV, capillary temperature set at 500°C, desolvation flow maintained at 1000 L/h, cone gas (nitrogen) flow at 150 L/h, and a cone voltage of 5 V. The collision energy and precursor to production transition *m*/*z* for each analyte are found in Table 1. The data acquisition was performed in multiple reaction monitoring (MRM) mode.

### 2.3. Standard Solutions and Quality Control Sample Preparation

Stock solutions of the eleven reference standards were prepared in methanol, with final concentrations of 2.92 mg/mL for coptisine chloride, 2.42 mg/mL for berberine, 2.65 mg/mL for columbamine, 1.50 mg/mL for phellodendrine, 1.91 mg/mL for palmatine, 5.01 mg/mL for obacunone, 4.56 mg/mL for esculetin, 4.92 mg/mL for fraxetin, 4.88 mg/mL for esculin, 5.03 mg/mL for fraxin, and 5.00 mg/mL for pulchinenoside B_4_. Each reference standard stock solution (1.0 mL) was combined and diluted with methanol to create a 100.0 mL standard mixture stock solution. Subsequently, a series of standard working solutions were generated by sequentially diluting the mixed stock solution with methanol. All working solutions were stored at 4°C in the dark.

Calibration standards were generated by introducing the standard working solutions into the blank plasma, resulting in final concentrations of 1.17 to 292.00 ng/mL for coptisine chloride, 0.97 to 242.00 ng/mL for berberine, 1.06 to 265.00 ng/mL for columbamine, 0.60 to 150.00 ng/mL for phellodendrine, 0.76 to 191.00 ng/mL for palmatine, 2.00 to 501.00 ng/mL for obacunone, 1.82 to 456.00 ng/mL for esculetin, 1.97 to 492.00 ng/mL for fraxetin, 1.95 to 488.00 ng/mL for esculin, 2.01 to 503.00 ng/mL for fraxin, and 2.00 to 500.00 ng/mL for pulchinenoside B_4_. Three levels of quality control (QC) samples (low, medium, and high) were prepared using the same methodology. All samples were stored at a temperature of −20°C.

### 2.4. Plasma Sample Preparation

Methanol and acetonitrile were compared in terms of their efficacy in protein precipitation, and acetonitrile was selected due to its superior extraction recovery. Subsequently, a volume of 400 *μ*L of acetonitrile was added to a 100 *μ*L plasma sample. The resulting mixture was subjected to vortexing for 1 minute and centrifuged at a force of 21367 g for 10 minutes at a temperature of 4°C. The resulting supernatant was subjected to evaporation, followed by reconstitution in a volume of 100 *μ*L of 10% acetonitrile. This reconstituted solution was then centrifuged at a force of 21367 g, and subsequently, a volume of 4 *μ*L of the resulting supernatant was used for UPLC-ESI-MS/MS analysis.

### 2.5. Method Validation

The evaluation of specificity involved the comparison of six separate blank plasma samples, blank plasma samples spiked with analytes, and plasma samples obtained after oral administration of BTWD.

Calibration curves were generated using weighed (1/*x*^2^) least-squares regression analysis, plotting the analyte peak areas (*y*) against the analyte concentrations in blank plasma (*x*). It was required that each calibration curve exhibit a correlation coefficient (*r*^2^) greater than 0.99. The determination of the lower limit of quantification (LLOQ) was based on the lowest concentration in the calibration curve that could be measured with acceptable precision and accuracy, within a range of ±15% for both parameters.

The precision and accuracy of the QC samples were assessed by analyzing eleven analytes in six replicates on the same day and on three separate days. The relative error (RE) and relative standard deviation (RSD) were computed.

Extraction recoveries of three QC levels were analyzed by comparing the peak area of analytes added to blank plasma before and after extraction. The matrix effect was evaluated by analyzing the peak area of the extracted blank plasma added to three QC concentration analytes and the corresponding analyte solutions.

The stability of both short-term and long-term conditions was assessed at room temperature for 24 hours and at −80°C for 10 days, respectively. Freeze-thaw cycle stability was evaluated by subjecting the samples to three cycles of freezing at −80°C and thawing at room temperature. Each test included the analysis of three quality control levels, with each level consisting of six samples. [14].

### 2.6. Pharmacokinetic Study

The study used six Bama miniature pigs (70 days old, weighing 20 ± 2 kg) obtained from the Experimental Animal Center, Shandong Lvdu Bio-Sciences and Technology Co., Ltd. (Binzhou, China). The pigs were housed under controlled conditions with a temperature of 25 ± 1°C, relative humidity of 65% ± 10%, and a 12/12 h light/dark cycle. The pigs were provided with standard pig feed and water ad libitum, in accordance with the guidelines outlined in the National Institutes of Health Guide for Care and Use of Laboratory Animals. Following a 12-hour fasting period, the pigs received BTWD intragastrically at a dose of 1.0 mL/kg, with approximate concentrations of coptisine chloride, berberine, columbamine, phellodendrine, palmatine, obacunone, esculetin, fraxetin, esculin, fraxin, and pulchinenoside B_4_ at 457.69, 3084.97, 542.65, 910.00, 736.02, 54.24, 751.43, 1537.92, 2810.50, 1943.49, and 3499.45 *μ*g/kg, respectively. Blood samples (0.5 mL each) were collected from the jugular vein of each pig into heparinized tubes at specific time intervals (0.5 h, 1.0 h, 4.0 h, 8.0 h, 12.0 h, 15.0 h, and 24.0 h) following intragastric administration. The blood sample was promptly subjected to centrifugation at a force of 3400 × g for 10 minutes. The resulting supernatant plasma was collected and preserved at a temperature of −80°C until subsequent preparation for LC-MS analysis. The concentration-time data of the analytes were evaluated using either noncompartmental or compartmental methods with the aid of the PKSolver 2.0 software package, and subsequently, pharmacokinetic parameters were computed. The outcomes are presented as the arithmetic mean accompanied by the standard deviation (SD).

## 3. Results and Discussion

### 3.1. Method Development

**Figure 1:**
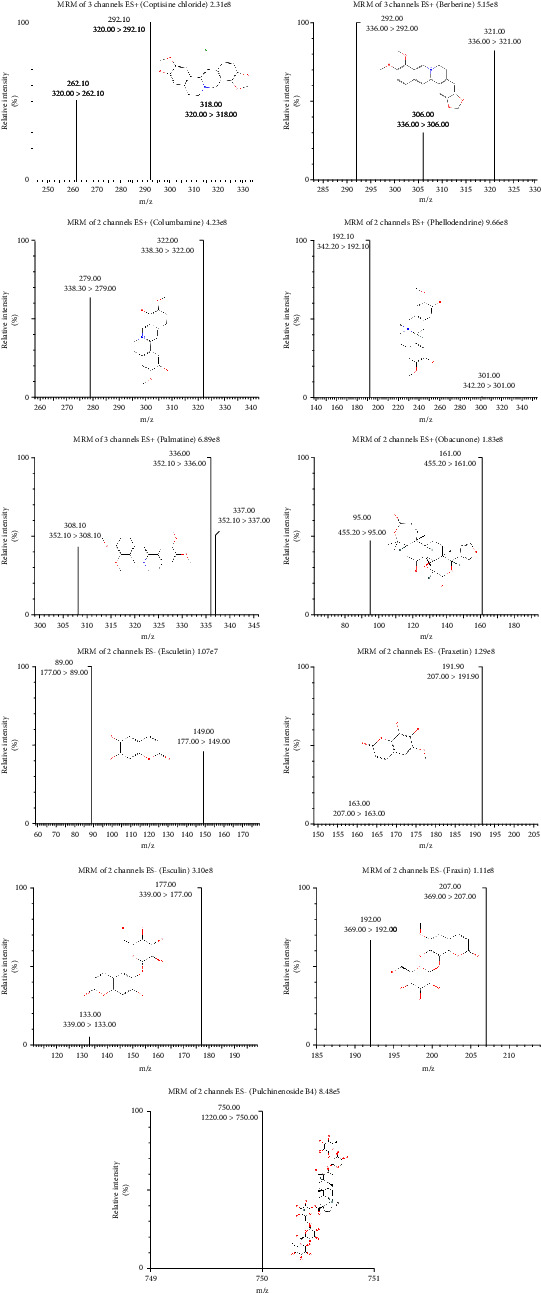
Product ion mass spectra and chemical structures of eleven analytes of BTWD.

Multiple reaction monitoring (MRM) was employed for the quantification of eleven analytes in pig plasma, thereby ensuring optimal peak shape and anticipated resolution. The optimized mass transition ion pairs (*m*/*z*) are delineated in Figure 1 and Table 1. To enhance peak responses and expedite analysis, a gradient elution of acetonitrile-water (0.1% formic acid) was selected. The findings demonstrated that all identified constituents were detected within a time frame of six minutes.

### 3.2. Method Validation

**Figure 2:**
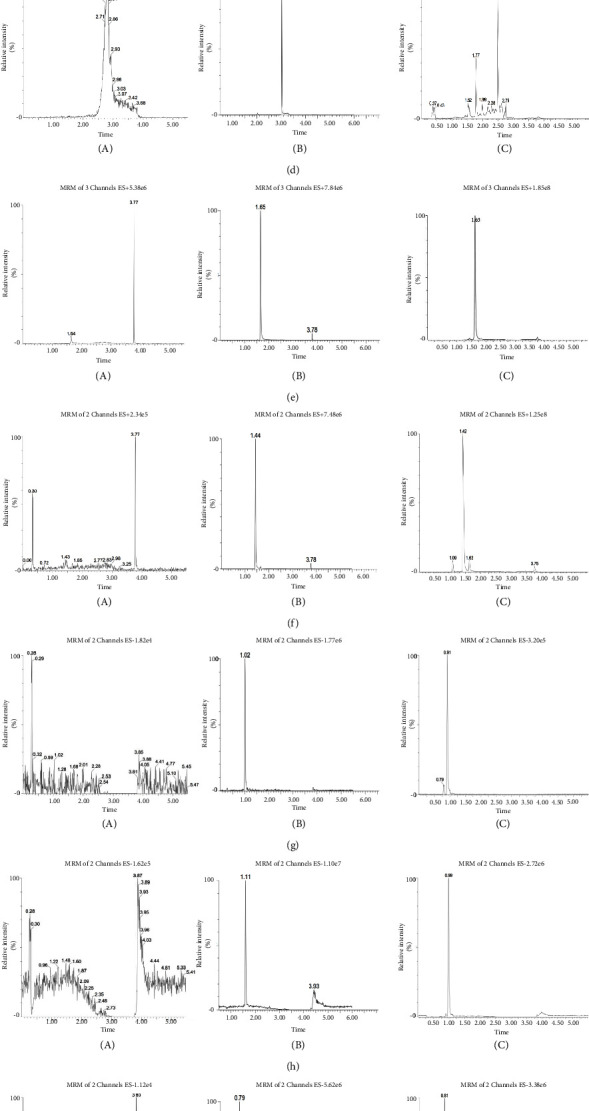
Representative MRM chromatograms of samples. 1, 2, and 3 in each chromatogram stand for blank plasma, blank plasma spiked with analytes at LLOQ, and processed samples at 0.5 h after oral administration of BTWD (1.0 mL/kg) in Bama miniature pigs, respectively. Letters (a–k) stand for different chemicals: (a) coptisine chloride, (b) phellodendrine, (c) palmatine, (d) obacunone, (e) berberine, (f) columbamine, (g) esculetin, (h) fraxetin, (i) esculin, (j) fraxin, and (k) pulchinenoside B_4_. The chromatographic separation was carried out on an Acquity UPLC HSS T3 C_18_ column with a gradient mobile phase consisting of acetonitrile and water (containing 0.1% acetic acid) at a flow rate of 0.4 mL/min. All analytes were quantitated through electrospray ionization in positive or negative ion multiple reaction monitoring (MRM) mode. The results showed that the retention time of all detected components was 6 mins. There were no apparent interferences for the eleven components.

Figure 2 displays chromatograms depicting blank plasma, plasma spiked with the analytes, and plasma obtained from a pig following oral administration of the BTWD extract. No discernible interferences were observed for the eleven analytes, indicating a high level of selectivity of the method for BTWD in plasma. The linearity and LLOQ are presented in Table 2. The calibration curves exhibited strong linearity, as evidenced by correlation coefficients ranging from 0.999 to 1. The LLOQs for coptisine chloride, berberine, columbamine, phellodendrine, palmatine, obacunone, esculetin, fraxetin, esculin, fraxin, and pulchinenoside B_4_ were determined to be 1.17, 0.97, 1.06, 0.60, 0.76, 2.00, 1.82, 1.97, 1.95, 2.01, and 2.00 ng/mL, respectively, which were deemed sufficient for the PK studies. In Table 3, the intraday and interday precisions ranged from 1.00% to 13.33% and 0.52% to 9.19%, respectively, while the accuracy ranged from −6.96% to 7.90% and −6.63% to 5.73%. These results conform to the acceptance criteria outlined in the bioanalytical method validation guidelines, indicating that the method employed was reproducible and accurate in detecting all analytes in pig plasma. As indicated in Table 3, the accuracy exhibited a range of −13.38% to 0.67%, −11.92% to −0.98%, and −13.67% to −2.48%, respectively, which provide evidence of satisfactory room temperature stability, long-term stability, and freeze-thaw stability. The extraction recoveries fell within the range of 83.62% to 98.76%, while the matrix effect ranged from 82.93% to 110.91%. These results demonstrate the effectiveness and efficiency of protein precipitation, as well as the negligible influence of the matrix on the detection of analytes in pig plasma.  Table 4 presents the detailed results.

### 3.3. Plasma Pharmacokinetics

**Figure 3:**
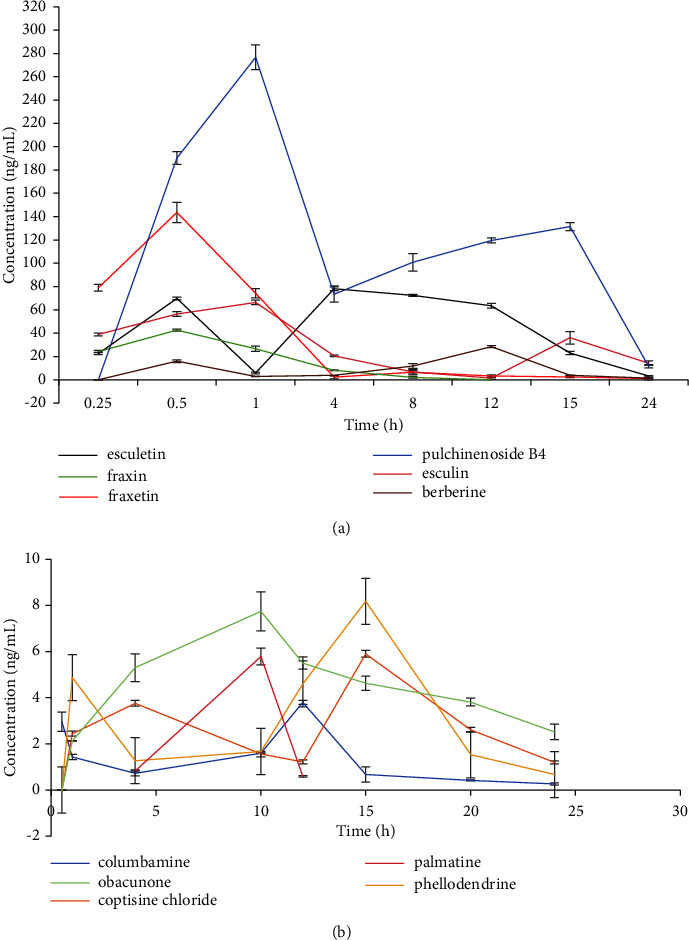
Concentration-time curves of 11 analytes in Bama miniature pig plasma after oral administration of BTWD (1.0 mL/kg). (a) Curves of esculetin, fraxetin, esculin, fraxin, pulchinenoside B_4_, and berberine; (b) curves of columbamine, coptisine chloride, phellodendrine, obacunone, and palmatine.

The validated method was used to assess the pharmacokinetics (PK) of coptisine chloride, berberine, columbamine, phellodendrine, palmatine, obacunone, esculetin, fraxetin, esculin, fraxin, and pulchinenoside B_4_ in pig plasma subsequent to a single oral administration of BTWD extract (1.0 mL/kg).  Figure 3 displays the plasma concentration-time profiles of the eleven analytes in pig plasma following oral administration. Noncompartmental methods were employed to analyze the concentration-time data of esculetin, esculin, coptisine chloride, phellodendrine, pulchinenoside B_4_, and berberine, whereas compartmental methods were used to analyze fraxetin, fraxin, columbamine, and obacunone in order to calculate the PK parameters, which are presented in Table 5.

The findings of the present study indicate that the alkaloid compounds berberine, columbamine, phellodendrine, and coptisine chloride exhibited peak concentrations in plasma at 12 hours. These compounds were found to have low plasma concentrations, with berberine demonstrating the highest maximum plasma concentration (*C*_max_) of 28.36 ± 0.83 ng/mL. On the contrary, columbamine, coptisine chloride, and phellodendrine demonstrated plasma concentrations below 10.0 ng/mL, indicating restricted absorption via the gastrointestinal tract. Typically, molecules must possess lipophilic properties to facilitate efficient absorption in the gastrointestinal tract. Conversely, polar molecules exhibit reduced lipophilicity. The polar nature and presence of ionic charges in the structures of these three molecules hinder their absorption in the gastrointestinal tract. Additionally, the plasma concentration of palmatine fell below the requisite threshold for the analysis of pharmacokinetic behavior, potentially attributable to its polar structure [12]. The plasma concentration of palmatine was found to be below the threshold required for the analysis of PK behavior. However, the concentration of palmatine at the 12-hour timepoint was determined to be 5.79 ng/mL, exhibiting variance from the documented profile of palmatine in rat and dog plasma. In particular, in rats, the *C*_max_ of palmatine was recorded as 2.14 ± 0.68 ng/mL and 2.50 ± 0.43 ng/mL, with the time to reach maximum concentration (*T*_max_) values of 0.36 ± 0.074 hours and 3.22 ± 0.81 hours following oral administration of Coptis root granules and Shuanghua Baihe tablets, respectively [19]. In beagle dogs, a *C*_max_ of 8 ng/mL and *T*_max_ of 5 hours were observed after oral administration of 300 mg of palmatine [20], indicating that the pharmacokinetic parameters of palmatine are influenced by coexisting compounds.

The *C*_max_ of esculetin, esculin, fraxetin, and fraxin was determined to be 78.13 ± 1.68, 66.43 ± 1.91, 118.75 ± 6.14, and 36.83 ± 0.48 ng/mL, respectively. The original concentrations of these compounds were measured to be 751.43, 2810.50, 1537.92, and 1943.49 ng/kg, respectively. These findings indicate that the *C*_max_ of fraxetin was higher than that of fraxin, and the *C*_max_ of esculetin was higher than that of esculin. This observation suggests that the conversion of esculin and fraxin into esculetin and fraxetin, respectively, may contribute to these differences in *C*_max_ values.

The *T*_max_ and *t*_1/2_ values of pulchinenoside B_4_ were determined to be 1.00 h and 3.27 ± 0.17 h, respectively, indicating rapid absorption and elimination from the plasma. In comparison with other compounds, pulchinenoside B_4_ exhibited the highest *C*_max_ of 276.70 ± 10.54 ng/mL and the largest AUC_0−*t*_ of 2525.63 ± 87.16 ng/mL·h, suggesting a high level of bioavailability.

On the contrary, the obacunone exhibited a low *C*_max_ of 7.39 ± 0.71 ng/mL, attributed to its low initial concentration. However, it demonstrated a prolonged *C*_max_ of 3.49 ± 0.07 h and MRT of 17.39 ± 3.00 h, contrasting with the previously reported *T*_max_ of 1–2 h and MRT of 4.30 ± 0.16 h in rats following oral administration of 10 mg/kg obacunone [21]. Additionally, compared to the *T*_max_ of 1.67 ± 0.29 h and MRT of 4.90 ± 2.60 h in rats administered with the fruit of *Tetradium ruticarpum* and licorice extracts together [22], the extended *T*_max_ and MRT of obacunone suggest that its pharmacokinetic behavior can be altered when used in combination with other drugs.

The concentration-time profiles of esculetin, fraxetin, esculin, columbamine, coptisine chloride, phellodendrine, pulchinenoside B_4_, and berberine displayed biphasic patterns, indicating the potential involvement of enterohepatic circulation, distribution re-absorption, or biotransformation [11]. The absorption of drugs is a multifaceted process influenced by interactions with various physicochemical and physiological factors. Factors such as the absorption window along the gastrointestinal tract, enterohepatic recirculation, variable gastric emptying, and drug-drug interactions can impact the absorption kinetics. Distribution re-absorption occurs when the drug concentration in tissue exceeds that in plasma, leading to the transfer of the drug from tissue to plasma and resulting in a secondary peak in plasma levels. For example, berberine, with its high concentration in bile during distribution, may facilitate enterohepatic circulation and distribution re-absorption [23]. The second peaks of esculetin and fraxetin may be caused by the esculin and fraxin biotransformation of their respective precursors, esculin and fraxin. The dual peak phenomena observed in these constituents may play a role in the sustained elevation of their blood concentrations *in vivo*, thereby enhancing the pharmacodynamic effects of BTWD [24].

In this study, the PK behaviors of pulchinenoside B_4_, phellodendrine, berberine, obacunone, esculin, and esculetin exhibited variations compared with the findings reported by Yang et al. [14, 16]. Similarly, fraxin demonstrated dissimilarities from the observations made by Wang et al. [13]. These disparities may be attributed to drug-drug interactions within the multiherbal mixture, leading to alterations in the PK parameters of the individual components [15]. Additionally, the use of different experimental animals could have contributed to these discrepancies. We assert that our results are more reliable as the animal model employed closely resembles that of humans.

## 4. Conclusion

This study presents the development and validation of a novel UHPLC-MS/MS method for the simultaneous quantification of eleven analytes in Bama miniature pig plasma. The method incorporates a straightforward plasma sample preparation technique. Rigorous validation procedures were conducted to assess the method's specificity, sensitivity, accuracy, and reproducibility. All validation parameters were found to meet the necessary bioanalysis criteria. Furthermore, the method was effectively used in pharmacokinetic studies of pigs following a single oral administration of 1.0 mL/kg BTWD. BTWD is commonly used in the management of digestive system disorders, with variations in its pharmacokinetic characteristics observed between normal and ulcerative colitis rats. Therefore, further investigation is warranted to assess the pharmacokinetic properties of BTWD compounds following administration to an ulcerative colitis model of Bama miniature pigs. Given the alkaloid compounds' low *C*_max_ and their significant therapeutic roles in digestive system diseases, they may be extracted separately and these compounds were administered through nonoral routes.

## Figures and Tables

**Table 1:** Mass spectrometry parameters of 11 analytes.

Analytes	Parent ion (*m*/*z*)	Daughter ion (*m*/*z*)	Collision energy (eV)	Retention time (min)
Coptisine chloride	320.0 (+)	292.1^*∗*^, 262, 318	27, 33, 30	1.49
Berberine	336.0 (+)	292^*∗*^, 306, 321	26, 28, 26	1.65
Columbamine	338.3 (+)	322^*∗*^, 279	26, 35	1.44
Phellodendrine	342.2 (+)	192.1^*∗*^, 301	20, 12	0.96
Palmatine	352.1 (+)	336^*∗*^, 308.1, 337	28, 25, 23	1.62
Obacunone	455.2 (+)	161^*∗*^, 95	27, 32	2.53
Esculetin	177.0 (−)	89^*∗*^, 149	22, 22	1.02
Fraxetin	207.0 (−)	191.9^*∗*^, 163	16, 17	1.11
Esculin	339.0 (−)	177^*∗*^, 133	25, 28	0.79
Fraxin	369.0 (−)	207^*∗*^, 192	19, 30	0.93
Pulchinenoside B_4_	1220 (−)	750^*∗*^	46	1.52

*Note*. ^*∗*^Means for quantification.

**Table 2:** The linear ranges, regression equations, and LLOQs for the determination of 11 components in pig plasma.

Analytes	Calibration curves	*R* ^2^	Ranges (ng/mL)	LLOQ (ng/mL)
Coptisine chloride	*y* = 1*E* − 05*x*–0.5584	0.9997	1.17–292.00	1.17
Berberine	*y* = 8*E* − 06*x*–2.5152	1	0.97–242.00	0.97
Columbamine	*y* = 1*E* − 05*x*–0.4069	0.9998	1.06–265.00	1.06
Phellodendrine	*y* = 4*E* − 06*x*–0.7192	0.9998	0.60–150.00	0.60
Palmatine	*y* = 6*E* − 06*x*–0.7511	0.9996	0.76–191.00	0.76
Obacunone	*y* = 0.0004*x*–2.0664	0.9996	2.00–501.00	2.00
Esculetin	*y* = 0.0025*x*–1.1961	0.9991	1.82–456.00	1.82
Fraxetin	*y* = 0.0013*x*–0.0661	0.9997	1.97–492.00	1.97
Esculin	*y* = 0.0003*x*–0.6909	0.9995	1.95–488.00	1.95
Fraxin	*y* = 0.0006*x*–2.6841	0.9996	2.01–503.00	2.01
Pulchinenoside B_4_	*y* = 0.1017*x*–7.9946	0.999	2.00–500.00	2.00

**Table 3:** The accuracy, precision, and stability of eleven ingredients of BTWD in pig plasma (*n* = 6).

Analytes	Spiked (ng/mL)	Intraday		Interday		Stability					
						Short term		Long term		Freeze-thaw cycles	
		RSD (%)	RE (%)	RSD (%)	RE (%)	RSD (%)	RE (%)	RSD (%)	RE (%)	RSD (%)	RE (%)
Coptisine chloride	4.67	3.83	−3.73	4.93	−1.30	1.53	−1.21	2.86	−4.11	2.67	−4.83
	11.68	3.93	−6.12	5.51	−2.83	2.67	−1.79	3.01	−6.17	3.10	−8.64
	116.80	3.71	5.53	5.31	1.81	0.53	−1.59	0.99	−2.31	3.98	−3.67

Berberine	3.87	5.43	−6.96	4.61	−5.06	1.11	−2.95	1.75	−4.82	1.33	−5.52
	9.68	3.57	5.10	0.52	5.00	1.47	−2.59	5.88	−6.33	4.72	−3.03
	96.80	1.65	−3.52	2.13	−2.55	5.00	−8.48	1.53	−6.64	2.78	−4.33

Columbamine	4.24	3.93	−0.99	3.64	0.33	2.26	−3.05	1.61	−1.83	3.61	−7.69
	10.60	2.64	−1.64	1.50	−0.85	2.08	−8.33	2.19	−2.94	2.36	−4.90
	106.00	4.82	−2.85	1.85	−2.81	2.44	−2.75	1.14	−3.68	3.22	−3.94

Phellodendrine	2.40	3.85	−1.78	8.92	4.62	2.70	−7.50	4.09	−6.67	4.17	−8.33
	6.00	3.63	−2.03	3.31	5.29	2.62	−5.71	1.04	−2.44	0.74	−3.57
	60.00	6.05	6.60	1.26	5.73	0.65	−1.85	1.03	−3.00	0.66	−2.96

Palmatine	3.06	13.33	7.90	4.87	1.55	5.42	−6.11	5.77	−7.22	4.81	−8.33
	7.64	3.85	−1.42	4.64	0.09	3.20	−4.67	1.19	−3.33	0.69	−4.00
	76.40	1.63	−5.88	2.03	−4.45	1.02	−4.61	2.88	−3.91	0.94	−5.00

Obacunone	8.02	3.66	−1.33	2.65	3.52	2.56	−10.50	2.33	−10.83	1.37	−7.83
	20.04	9.20	1.36	9.19	4.92	0.75	−1.15	0.64	−1.79	0.81	−2.48
	200.40	4.04	0.45	1.92	−1.90	2.86	−5.52	1.52	−7.53	1.75	−5.21

Esculetin	7.30	1.24	3.90	2.77	4.98	2.14	−2.67	2.84	−11.33	1.77	−13.67
	18.24	1.00	2.02	0.81	−0.31	1.21	−1.67	1.68	−3.73	2.86	−4.31
	182.40	3.01	1.69	7.13	3.48	0.59	−3.55	4.82	−7.52	2.80	−4.41

Fraxetin	7.87	6.54	−4.34	1.21	−2.19	0.87	−5.05	2.66	−7.14	2.30	−5.24
	19.68	3.11	6.98	4.12	3.05	1.28	−8.22	1.19	−8.50	1.21	−10.25
	196.80	3.32	−0.65	2.33	2.16	5.93	−8.10	1.10	−5.99	1.38	−6.82

Esculin	7.81	5.12	−1.75	3.06	−0.30	0.74	−11.22	1.39	−9.22	5.33	−11.82
	19.52	3.92	4.75	3.64	1.01	0.40	−1.83	3.27	−3.50	0.65	−4.29
	195.20	2.35	3.16	2.50	3.64	0.49	−3.38	2.94	−5.45	0.79	−5.52

Fraxin	8.05	3.69	1.49	3.92	2.94	6.68	−1.82	3.08	−3.58	1.94	−5.12
	20.12	4.96	1.71	4.11	−0.31	1.42	−0.90	2.30	−5.24	3.07	−6.75
	201.20	2.82	2.75	6.67	3.19	6.37	0.67	3.31	−0.98	0.84	−2.87

Pulchinenoside B_4_	8.00	5.54	3.29	5.85	4.62	0.80	−13.38	6.43	−11.92	5.49	−10.00
	20.00	1.91	−4.55	2.03	−6.63	1.27	−7.77	0.42	−8.53	0.93	−8.78
	200.00	3.76	1.04	1.65	−2.62	0.64	−5.76	3.22	−7.98	2.87	−8.66

**Table 4:** Extraction recovery and matrix effect of eleven ingredients in pig plasma (*n* = 6).

Analytes	Extraction recovery (%)			Matrix effect (%)		
	Low	Medium	High	Low	Medium	High
Coptisine chloride	93.00 ± 1.40	86.70 ± 5.87	92.42 ± 2.32	105.00 ± 6.24	95.50 ± 1.25	93.48 ± 1.65
Berberine	86.65 ± 2.48	85.54 ± 1.26	92.43 ± 0.95	87.94 ± 4.46	85.74 ± 2.27	95.32 ± 10.69
Columbamine	94.50 ± 2.84	83.62 ± 0.94	91.29 ± 2.76	100.24 ± 2.27	91.79 ± 3.02	103.94 ± 1.78
Phellodendrine	91.04 ± 1.39	90.91 ± 7.93	88.22 ± 4.02	103.74 ± 5.16	110.91 ± 1.82	89.33 ± 4.37
Palmatine	92.78 ± 4.19	90.89 ± 1.39	95.39 ± 0.97	101.67 ± 4.41	87.78 ± 2.69	93.67 ± 0.69
Obacunone	87.17 ± 2.84	91.56 ± 3.83	92.72 ± 2.51	104.33 ± 1.53	98.21 ± 0.89	95.88 ± 0.59
Esculetin	92.72 ± 0.66	93.43 ± 1.86	89.42 ± 1.63	101.77 ± 0.38	97.71 ± 2.32	94.55 ± 0.91
Fraxetin	95.55 ± 0.46	98.76 ± 0.24	95.39 ± 1.75	100.76 ± 0.13	99.29 ± 0.22	95.61 ± 0.30
Esculin	88.31 ± 0.27	92.83 ± 0.10	93.60 ± 0.75	103.84 ± 0.22	95.13 ± 0.05	98.29 ± 0.30
Fraxin	92.63 ± 6.10	91.48 ± 5.00	96.24 ± 6.37	93.04 ± 6.51	87.64 ± 5.37	92.91 ± 0.89
Pulchinenoside B_4_	92.13 ± 2.21	90.33 ± 1.54	92.86 ± 1.13	109.00 ± 1.19	82.93 ± 0.67	94.65 ± 0.29

**Table 5:** Pharmacokinetic parameters of 10 components of BTWD after oral administration in pigs (*n* = 6).

Analytes	*C*_max_ (ng/mL)	*T*_max_ (h)	*t*_1/2_ (h)	CL/F (mL/h)	AUC_0−*t*_ (ng/mL·h)	AUC_0− inf_ (ng/mL·h)	MRT (h)
Esculetin	78.17 ± 1.68	4.00	2.91 ± 0.08	0.76 ± 0.02	980.37 ± 26.44	994.38 ± 27.79	8.97 ± 0.08
Esculin	66.43 ± 1.91	1.00	16.63 ± 1.48	3.25 ± 0.43	531.20 ± 52.83	875.86 ± 124.12	25.06 ± 1.91
Fraxetin	118.75 ± 6.14	0.49 ± 0.01	0.33	9.72 ± 0.56	158.57 ± 9.33	158.57 ± 9.33	0.98 ± 0.01
Fraxin	36.83 ± 0.48	0.53 ± 0.02	0.36 ± 0.01	36.38 ± 1.84	53.25 ± 2.70	53.51 ± 2.78	1.07 ± 0.04
Columbamine	3.80 ± 0.08	12.00	6.74 ± 5.08	17.54 ± 0.76	28.35 ± 1.31	30.98 ± 1.36	12.50 ± 2.80
Coptisine chloride	5.90 ± 0.15	12.00	5.70 ± 0.33	6.56 ± 0.14	59.88 ± 0.15	69.80 ± 1.42	14.34 ± 0.31
Berberine	28.36 ± 0.83	12.00	3.14 ± 0.13	14.82 ± 0.93	202.44 ± 12.74	208.64 ± 13.59	11.07 ± 0.03
Phellodendrine	8.18 ± 0.21	12.00	3.81 ± 0.10	12.41 ± 0.47	69.72 ± 2.62	73.39 ± 2.80	11.39 ± 0.14
Obacunone	7.39 ± 0.71	3.49 ± 0.07	0.88 ± 0.15	0.37 ± 0.02	110.70 ± 3.90	1146.51 ± 7.38	17.39 ± 3.00
Pulchinenoside B_4_	276.70 ± 10.54	1.00	3.27 ± 0.17	1.36 ± 0.05	2525.63 ± 87.16	2580.65 ± 89.09	9.57 ± 0.14

## Data Availability

The data used to support the findings of this study are included within the article.

## Supplementary Materials

Supplementary Materials Chromatogram of 11 components, which contain coptisine chloride, berberine, columbamine, phellodendrine, palmatine, obacunone, esculetin, fraxetin, esculin, fraxin, and pulchinenoside B_4_.
