# Supplementary figures and images for: Pharmacokinetic Properties of Baitouweng Decoction in Bama Miniature Pigs: Implications for Clinical Application in Humans
Source: Int J Anal Chem. 2024 May 10;2024:5535752. doi: 10.1155/2024/5535752 (PMC11101253; doi:10.1155/2024/5535752)

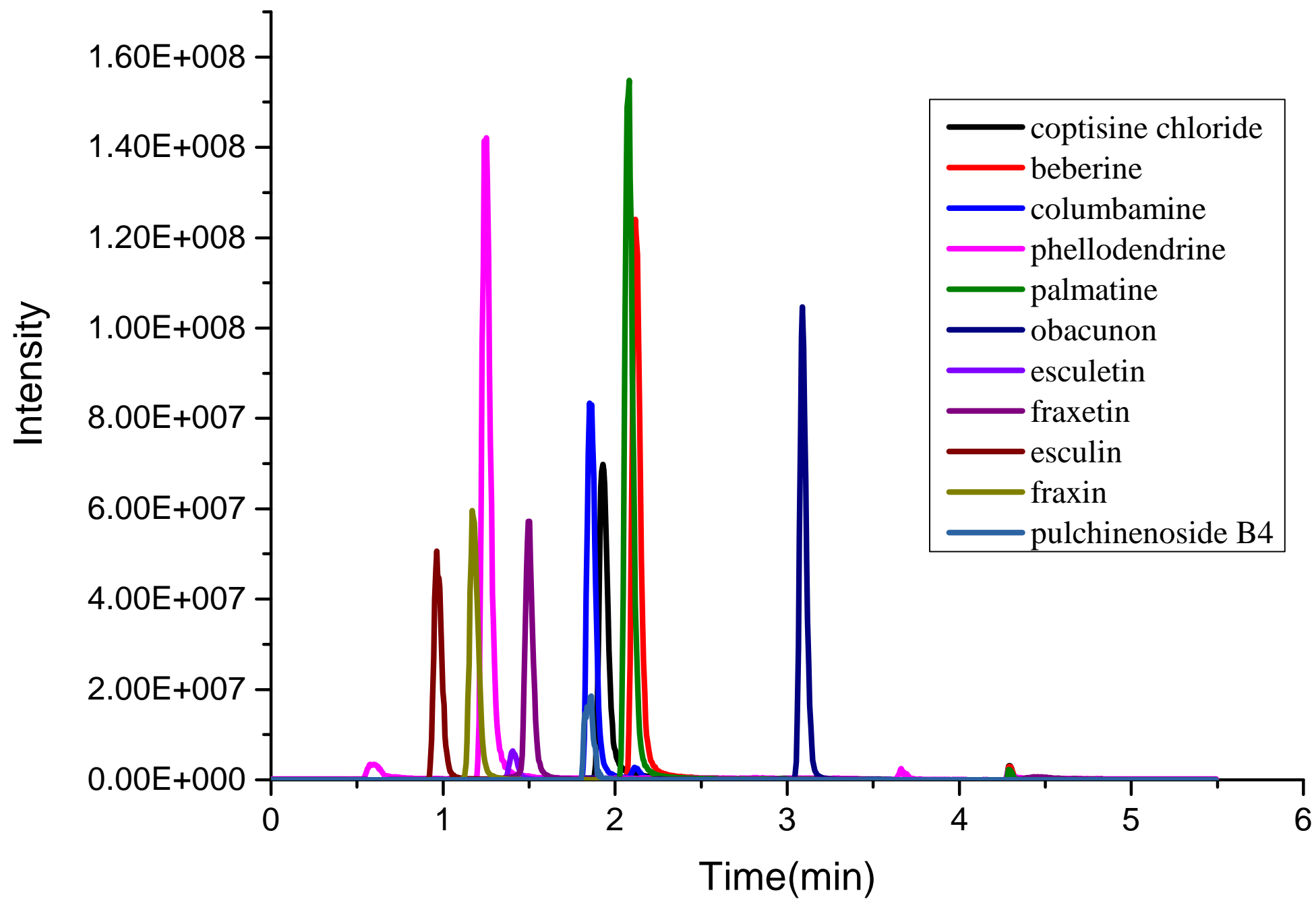

Supplement: Supplementary Materials — Chromatogram of 11 components, which contain coptisine chloride, berberine, columbamine, phellodendrine, palmatine, obacunone, esculetin, fraxetin, esculin, fraxin, and pulchinenoside B4. [file 5535752.f1.pdf]
